# Ambulance clinicians’ perspectives of sharing patient information electronically

**DOI:** 10.29045/14784726.2019.12.4.3.49

**Published:** 2019-12-01

**Authors:** Jack W. Barrett

**Affiliations:** South East Coast Ambulance Service NHS Foundation Trust

## Abstract

**Introduction:**

Communication in the NHS is vital to patient care and safety. Government bodies are pushing for the digitisation of patient health records so that access and transfer of information is easier between patient care teams. Many ambulance trusts have issued their clinical staff tablet computers as a step in the transition from paper-based to electronic-based patient health records. This study aims to evaluate whether these ambulance clinicians perceive tangible benefits to digitisation, particularly regarding collaborative working with other healthcare professionals.

**Methods:**

Registered and non-registered clinical staff in one ambulance trust completed an online questionnaire utilising five-point Likert scales to collect data about their experiences of using electronic incident summary notifications to report back to the patient’s GP, and on direct patient referrals to community teams for falls and hypoglycaemic episodes. Participants only completed questions relevant to the process they had experienced.

**Results:**

From approximately 2115 members of staff eligible to participate, there were 201 respondents (9.50%) who provided information concerning GP summary notifications, fall referrals or hypoglycaemia referrals (n = 154, 76.62%; n = 178, 88.56%; n = 101, 50.25%, respectively).

Overall, staff perceived the electronic communication of patient information as useful, but not essential, to their practice. The applications were seen as easy to use and a safer way to handle patient data. Though their use was felt to prolong the time spent on scene, this was regarded as an efficient use of a clinician’s time.

Many staff would prefer to talk directly to a patient’s GP, but fewer felt that this was required for community referrals. While most participants did not feel obliged to send a GP summary notification of every encounter, the majority believed that the rates of appropriate falls and hypoglycaemia referrals would be improved with direct electronic communication.

Respondents felt that recording and sharing patient information electronically improved collaborative working with other healthcare professionals, and they preferred having this ability.

**Conclusion:**

NHS ambulance trusts are transitioning to electronic patient records and this article suggests that ambulance staff are in favour of this transition when the technology is readily accessible and easy to use. Staff believe this approach is a safer way to store and share patient data and that collaborative working is enhanced. However, many clinicians would still prefer to discuss some incidents directly with a GP rather than sending a summary, highlighting the value staff place on real-time professional interaction when managing a patient.

